# Post-Treatment Lyme Disease Syndrome: Need for Diagnosis and Treatment

**DOI:** 10.7759/cureus.18703

**Published:** 2021-10-12

**Authors:** Simona Maksimyan, Munir S Syed, Varun Soti

**Affiliations:** 1 Infectious Disease, Lake Erie College of Osteopathic Medicine, Elmira, USA; 2 Pathology and Histology, Lake Erie College of Osteopathic Medicine, Elmira, USA; 3 Pharmacology and Therapeutics, Lake Erie College of Osteopathic Medicine, Elmira, USA

**Keywords:** treatment strategies, diagnostic criterion, standardized treatment, antibiotic, chronic lyme disease

## Abstract

With the continued surge in Lyme disease cases, post-treatment Lyme disease syndrome (PTLDS) is becoming a more pressing health concern. The aim of this review is to identify comprehensive treatment strategies for PTLDS patients. Unfortunately, universal guidelines for diagnosing and treating PTLDS do not currently exist. Consequently, physicians cannot adequately address concerns of possible PTLDS patients. Patients are left suffering and searching for answers, and their activities of daily living and quality of life are adversely impacted. This review highlights that PTLDS clinical trials have focused mainly on treatment with antibiotics, yielding challenging results that lack consistency in inclusion criteria across trials. It will remain exceedingly difficult to extrapolate the outcomes of such studies if a standard for PTLDS diagnosis is not well-established. By focusing on treatment trials rather than establishing diagnostic criteria, research in this field ignores a critical step in investigating PTLDS. The first significant step is to create comprehensive guidelines for the diagnosis of PTLDS, which can generate uniformity and validate PTLDS treatment trials.

## Introduction and background

The incidence of Lyme disease (LD) has been progressively increasing, consequentially escalating the importance of research on the diagnosis and treatment of post-treatment Lyme disease syndrome (PTLDS). It was estimated that between 2010 to 2018, there was an average of 476,000 LD diagnoses per year in the United States (US). This number is far greater than the 329,000 cases per year estimated from 2005 to 2010 and indicates an increase in the number of cases over time. Furthermore, 81% percent of LD cases from 2010 to 2018 occurred in 14 states located in the northeast, mid-Atlantic, and upper Midwest regions, where incidence rates are high [1]. This data has not been updated since 2018.

LD is a vector-borne disease, commonly caused by the spirochetal bacterium *Borrelia (B.)* discovered in 1981 in an Ixodes scapularis tick [2]. As LD cases continue to rise, using a standardized diagnosis and treatment method is imperative. A two-step testing approach for diagnosis and the use of antibiotics for the treatment of LD has long been a national standard in the US, as set forth by the Centers for Disease Control and Prevention (CDC), with agreement from the National Institute of Allergy and Infectious Diseases (NIAID). However, a proper diagnosis and treatment regimen for chronic LD, also referred to as PTLDS, has not been standardized. In PTLDS, some symptoms of LD persist beyond initial therapy in the acute phase, and with the growing number of LD cases, PTLDS is becoming a more significant concern. This review highlights the need for proper diagnosis, existing treatment regimens, and their efficacy in managing PTLDS.

PTLDS is a condition in which patients experience symptoms of fatigue, pain, and cognitive difficulties that persist past the initial antibiotic treatment of acute LD, lasting over six months post-treatment [3]. Chronic LD and PTLDS will be used interchangeably throughout this review, as there is currently no established difference between the two. Although the criteria for LD have been set, the CDC does not now provide guidelines for PTLDS. The lack of universal diagnostic criteria has made the number of cases of PTLDS extremely difficult to estimate at this point in time. Nonetheless, the number of LD diagnoses has been rising, and as the population of previous and current LD patients grows, so does the number of people at risk for developing PTLDS. The need for research in this field is based on the same reasoning as most other chronic conditions: patients with PTLDS live with symptoms that affect their day-to-day lives, and the lack of evidence-based diagnostic and treatment procedures is a roadblock to improving their situation.

While PTLDS has been formally recognized by the CDC and the NIAID, the medical community has a scarcity of credible and comprehensive information and education on this condition. As long as this is the case, physicians will not effectively address patients with PTLDS, and patients will continue to suffer without answers or proper treatment. This review focuses on the necessity for uniform guidelines for the diagnosis and standardized treatment regimens for PTLDS patients.

## Review

**Figure 1:**
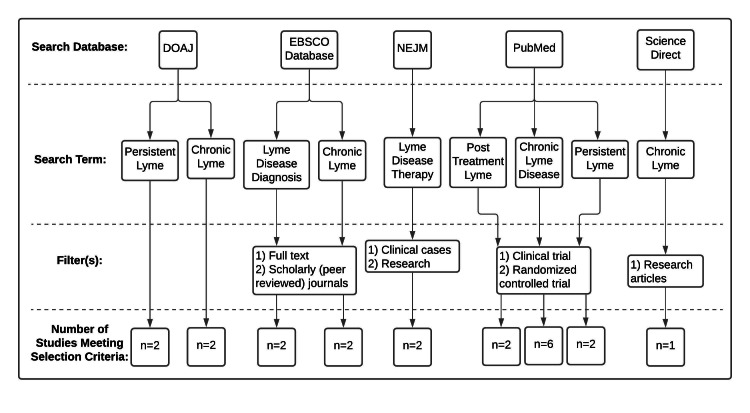
Article search process and the number of studies included This review utilized five databases to search the literature on post-treatment Lyme disease syndrome (PTLDS). The keywords were limited to two to three words to yield the largest variety of studies. By using filters and the inclusion criteria, the number of studies was narrowed to 21. DOAJ, Directory of Open Access Journals; EBSCO, Elton B. Stephens Company; NEJM, The New England Journal of Medicine; PTLDS, Post-treatment Lyme Disease Syndrome.

We conducted this literature review from February 2021 through June 2021. The inclusion criteria for the scientific papers included in this review were: (1) peer-reviewed; (2) clinical studies, retrospective studies, or laboratory studies. Only studies published in the English language were selected. The review focused on the possible symptomology of and treatment courses for PTLDS in the human model. The process of the literature search for the review section is shown in Figure 1. Twenty-one scientific papers were cited in the review section. Our conclusions were drawn only from these studies. Therefore, a possible limitation of this paper would be the exclusion of data and information from studies that did not meet the inclusion criteria and/or were not within the scope of this review.

Despair of patients with PTLDS

PTLDS patients are more likely to have impaired work, social, or family interactions [4]. These patients have diminished well-being and health-related quality of life comparable to people with other chronic illnesses [5]. Even though PTLDS is a severe and possibly chronic illness that is likely to become more familiar with the growing number of LD cases, relatively little is known about it. Researchers have not been able to identify why PTLDS occurs, what its predisposing factors are, and most importantly, how to diagnose, treat, and prevent it. As a result, patients, as well as physicians, have minimal guidance, which creates room for error.

Due to a lack of effective treatment options, PTLDS patients may turn to non-credible sources, which prey on their fears and desperation, for answers and help. A study published in 2015 found over 30 alternative LD therapies via Google search engine, including ultraviolet light, urine ingestion, bee venom, and stem cell transplantation, none of which are supported by evidence-based science [6]. Physicians are equally challenged by the lack of PTLDS information, as revealed by a 2006 study in Connecticut, a known endemic area, in which 48.1% of primary care physicians surveyed were undecided about the existence of chronic LD, 49.8% did not believe chronic LD existed. A mere 2.1% had previously diagnosed and treated chronic LD [7].

Lack of standardized antibiotic treatment regimen used to manage patients with PTLDS

Long-term trials of antibiotics are the most thoroughly studied treatment regimens for PTLDS. However, it is worth noting that the specific antibiotics used, the length of treatment, the symptoms being treated, and the outcomes vary amongst these studies.

Several studies on chronic LD have noted cognitive deficits as a commonly reported symptom, likely because cognitive deficits can have a pronounced impact on a person’s ability to perform their activities of daily living (ADLs). Although symptoms of cognitive deficits are typically self-reported and may not be objectively tested in every case, it is essential to remember that in the review of chronic LD symptoms, the patient’s perception of their own illness may not always be supported by objective measures. Nevertheless, the presence of cognitive deficits, whether subjective or objective, can heavily influence a patient’s life and their ability to function in their respective environments.

The Johns Hopkins Lyme Disease Research Center used the CDC’s 2011 criteria to recruit 124 participants with a history of LD who began experiencing fatigue, musculoskeletal pain, and/or neurocognitive complaints within six months of their diagnosis. Ninety-two percent (92%) of participants reported cognitive complaints; only 26% were found to have cognitive decline through neuropsychological evaluation while 50% did not meet the measures for cognitive decline. Patients with definitive cognitive decline performed significantly worse on processing speed and verbal learning and memory, determined with a nonstructured word list-listening task. Interestingly, 24% of participants could not be included in the objective testing data due to suboptimal engagement during testing [8]. Although most participants self-reported neurocognitive deficits, at least half of the participants had no objective deficits, suggesting that a patient’s experiences can indeed be vastly different from a physician’s factual findings.

Additionally, this study exposes an important sub-group of PTLDS patients - those who could not adequately engage in tasks and tests that required their full attention and effort. Excluding this population may have skewed the results of this study while simultaneously uncovering an additional symptom of PTLDS. Further evaluation of such patients would likely provide important information about the attention and engagement capabilities of PTLDS patients.

Studies have exposed that PTLDS may be challenging to differentiate from neuropsychological disorders due to patients’ neurocognitive symptoms. A study comparing patients with PTLDS to patients with major depressive disorder (MDD) found that PTLDS patients perform significantly worse on memory tasks and are less depressed than MDD patients. Both groups presented with similar amounts of psychomotor slowing. Difficulty with language fluency was also more common in the PTLDS group, likely in close association with memory deficits, while depression was decreased. Thus, the investigators suggest that PTLDS patients will have a higher level of memory deficits and language difficulty along with less depression in comparison to MDD patients [9]. These findings can help physicians distinguish between PTLDS and MDD, as they may present in a very similar manner. It cannot be assumed that the presence and/or severity of depression in PTLDS patients is directly correlated with neurocognitive symptoms.

In the years 1997 to 2000, a study recruited participants in the northeast region of the United States who had been previously diagnosed with LD and had persistent symptoms following treatment. Patients were split into two groups: seropositive by Western blot for *B. burgdorferi* immunoglobulin (Ig) G antibodies and seronegative but with erythema migrans lesion history. Each group was further divided into two groups: an experimental group that received 2 grams of intravenous ceftriaxone per day for 30 days followed by 100 milligrams (mg) three times a day of oral doxycycline for 60 days, and a placebo group. Cognitive functioning, pain, role functioning scales, memory, and attention were not significantly different between groups at baseline. One-hundred percent (100%) of participants reported impaired cognition at baseline, but they had normal baseline neurophysiological test results. After completing the treatment course, all groups improved in the areas mentioned earlier without significant difference between the treatment and placebo groups, resulting in a lack of evidence for additional antibiotic therapy in the treatment of PTLDS. The investigators attributed this improvement in symptoms, which lasted at least three months, to practice-effect, less pain, and improved mood [10]. It is evident that self-reported symptoms do not always correlate to objective testing and may not be responsive to antibiotic therapy. In evaluating patients with possible chronic LD, especially when a persistent infection is not present, it is critical to use objective diagnostic means.

A European study, called Persistent Lyme Empiric Antibiotic Study Europe (PLEASE), recruited participants from 2010 to 2013 and assessed whether longer-term antibiotic treatment led to better outcomes in treating persistent symptoms attributed to LD. Persistent symptoms included musculoskeletal pain, arthritis, arthralgia, neuralgia, sensory disturbances, neuro-psychological problems, cognitive disorders, and fatigue and must have been accompanied by erythema migrans rash or *B. burgdorferi* IgG/IgM antibodies. Patient outcomes were measured using the RAND-36 Health Status Inventory (RAND SF-36) to assess the health-related quality of life. Participants were all given open-label intravenous ceftriaxone for two weeks, followed by blinded 12-week follow-up treatment with (1) doxycycline, (2) clarithromycin-hydroxychloroquine, or (3) placebo [11]. All groups showed improvement in health-related quality of life after initial treatment with ceftriaxone. However, no further significant improvement occurred during the second treatment for any group [12].

Cognitive performance was also evaluated in the aforementioned study, and no significant difference was found between the longer-term treatment and placebo groups [13]. This study concluded that longer-term antibiotic treatment regimens for persistent symptoms of LD had no additional benefits than a two-week course of antibiotics, in this case, ceftriaxone. A significant limitation of this study was its external validity to areas outside of Europe; the study did not require serological testing for all participants even though several different Borrelia strains may cause LD, especially between Europe and North America. Therefore, these findings should be applied to patients outside of Europe with caution.

A North American study conducted in the late 1990s in Suffolk County, Long Island, an area known to have increased tick populations, aimed to determine whether post-LD symptoms, specifically severe fatigue and slower mental speed, would be improved with long-term antibiotic therapy. The treatment group received 28 days of intravenous ceftriaxone and displayed a treatment benefit for fatigue compared to the placebo group. However, neither group had a treatment benefit for mental speed [14]. It is well demonstrated here that antibiotics would only improve some symptoms and might not be optimal for every patient. Hence, a cost-benefit analysis of long-term antibiotic use should be considered for individual patients.

From the late 1980s through the mid-1990s, a chronic LD study recruited participants in Connecticut and Massachusetts. Chronic LD was defined as having at least two of three symptom sets (fatigue, neurological complaints, or musculoskeletal complaints) persisting for over three months. Known tick bite or erythema migrans rash was not required although, 29% had a known tick bite and 44% had a known rash. Enzyme-linked immunosorbent assay (EIA) or western immunoblotting was positive for *B. burgdorferi* in 81% of participants (29% positive for EIA and 81% positive for western immunoblotting). The treatment regimen included 500 mg of tetracycline hydrochloride three times a day for at least one month, administered until the patient’s symptoms resolved or displayed significant improvement. The length of treatment ranged from one to 11 months, with a median of four months. Successful treatment in this study was labeled as improvement in symptoms or cure, in other words, the total absence of symptoms for one or more years post-treatment. The study found that in 80-90% of patients, a three to six-month course of treatment was associated with a cure or significant improvement in symptoms. It was noted that 22% of participants seropositive for *B. burgdorferi *became seronegative at the end of treatment. Perhaps the most important conclusion from this study is the positive, direct correlation between symptom duration and the duration of treatment required to achieve significant improvement or cure [15].

Although this study presents appealing results, it is crucial to keep in mind that the equivocal duration of treatment presents its own challenges. Applying this treatment regimen in practice may lead to antibiotic use for long and unknown periods, putting the patient at risk of adverse events. Henceforth, in addition to determining whether antibiotic treatment is appropriate for late LD, it must also be determined how long the course of treatment should be. While some studies may use an open-ended approach, administering treatment until a significant decrease in symptoms or cure is reached, other studies have tested specific short and long courses of treatment. What is considered short versus long is subjective, and various studies have defined this in different ways.

A US study conducted in the early to mid-1990s recruited participants with exposure to endemic areas and/or a history of an erythema migrans rash three months before the study and positive EIA and Western blot. These patients had objectively defined neurologic, rheumatologic, dermatologic, and arthritic issues and were split into two groups to be treated with either a 14-day or a 28-day course of intramuscular or intravenous ceftriaxone. Both groups experienced impressive overall cure rates, 76% and 70%, respectively, yet there was no significant difference in cure rates between them. Though not significantly different between the groups, there were five failures, meaning no evidence of response to therapy, in the 14-day group and zero failures in the 28-day group. Of note, cure rates were higher at the 12-month follow-up than at the three-month follow-up, implying continued improvement post-treatment. Thirteen total patients withdrew due to adverse events, 10 being in the 28-day group. Overall, the study determined that a 14-day course of ceftriaxone is an effective treatment for most late LD patients [16]. This study did not establish specific traits of patients who responded to treatment, making it difficult to decide which patients may benefit from a course of antibiotics and whether the risk of adverse events is worthwhile. In addition, the investigators did not specify whether participants were treated for LD in the acute phase, presenting a possible confounding factor.

Later studies tested even longer courses of antibiotic therapy, for example, comparing a 30-day course of intravenous ceftriaxone followed by 60 days of oral doxycycline within a group of seropositive and a group of seronegative patients presenting with persistent symptoms of LD. The first 107 participants to complete the study displayed no significant difference in treatment outcome between the Placebo and Treatment groups in either serological category. The study was halted from recruiting further participants due to the lack of efficacy of the antibiotic treatment. However, the Placebo group provided some information regarding the possible course of LD symptoms that persisted after acute-phase treatment. Thirty-six percent (36%) of the Placebo group improved in health status, 39% worsened, and 25% had no significant change throughout the study [17].

The impact of antibiotic-associated adverse effects on patients with PTLDS

Examples of adverse effects related to prolonged antibiotic use in LD have been shown in several studies. The PLEASE study reported many adverse events associated with the antibiotic treatment regimen, with 73.2% of patients reporting an adverse event. The most common events were rash, diarrhea, and allergic reactions, and only 6.8% of patients had to discontinue participation [12]. These symptoms lie on one end of the spectrum. A case report about a 59-year-old female treated for LD tells a different side of the story. This patient sought care for symptoms, including fatigue, insomnia, achy joints, memory loss, and confusion. Laboratory tests determined that she was IgM-positive but IgG-negative for *B. burgdorferi*, and she was treated with a five-week course of doxycycline for “possible LD.” Her symptoms improved initially but began to worsen after the antibiotic course was finished. The patient continued to seek medical help and, even though her primary care physician and rheumatologist suggested that there was no objective evidence of LD, another physician diagnosed her with chronic LD. She was prescribed oral cefuroxime and telithromycin for an additional two to four months. The patient eventually had adverse events and was diagnosed with a *Clostridium difficile *infection. She was treated with oral metronidazole, and the adverse events continued, eventually leading to death [18].

This case demonstrates the grave consequences of prolonged antibiotic use and the dangers of inappropriate diagnosis and lack of communication between members of a patient’s healthcare team. It also highlights the difficulties patients with these symptoms face when seeking care and answers about their chronic and perplexing medical conditions.

A retrospective cohort analysis of commercial health claims from 2013 to 2015 found significantly higher rates of adverse events in PTLDS patients being treated than those not treated. All-cause inpatient stays, emergency department visits, electrolyte imbalances, and infections within 90 days of PTLDS treatment with oral antibiotics, intravenous antibiotics, or immunomodulators were significantly higher than in the no-treatment comparison group. It is noteworthy that at the time of this study, there was neither a universal standard for diagnosis and treatment of PTLDS nor a specific diagnostic code for PTLDS. Therefore, this study might not have enrolled patients who were actually diagnosed with PTLDS [19].

Undermining of PTLDS prevalence due to lack of standardized diagnostic criteria

A highly relevant but challenging question comes into play regarding the epidemiology of PTLDS. What percentage of all patients diagnosed with early LD and treated with antibiotics go on to develop PTLDS? One study followed 63 patients in the United States mid-Atlantic suburban community from their initial diagnosis of early LD to their follow-ups after a three-week course of doxycycline. Follow-ups occurred after treatment and at one month, three months, and six months after completing antibiotic therapy. Symptoms of acute LD, including fever and chills, significantly decreased directly after treatment and reached nearly 0% at subsequent follow-ups. However, complaints of new-onset fatigue, widespread pain, and neurocognitive difficulties gradually increased during treatment and were recorded for 20% to 45% of patients during follow-ups. The study ultimately classified 35% of participants as having PTLDS, reporting that these patients have significantly lower life functioning within six months of treatment. These symptoms were noted at the follow-up immediately following treatment, so it is imperative to assess any residual symptoms that patients are experiencing after treatment, as they could indicate PTLDS in the future [20]. This study specifically recruited patients with early-diagnosed LD who received immediate treatment; however, not all cases of LD are diagnosed at the same time in the disease course, and it is rarely possible to determine the exact time of infection. This, along with many other confounding factors, makes predicting the likelihood of developing PTLDS very difficult. Also, these results come from a relatively small group of people from the same area, whose environment, socioeconomic status, and accessibility to healthcare, to name a few, may decrease this study’s external validity.

Looking beyond antibiotics for PTLDS management

The uncertainty surrounding the correct diagnosis and treatment of chronic LD has expectedly resulted in the exploration of forms of treatment other than antibiotic therapy. Disulfiram, a drug typically used to treat alcohol dependence, has been proposed as a treatment for late forms of Lyme disease. A recent questionnaire-based study conducted in France yielded 16 participants who were treated for late LD with disulfiram, with or without antibiotic adjunct therapy. Severe fatigue, joint pain, and cognitive issues were amongst the most common symptoms reported. Overall, 81.25% reported disulfiram-induced toxic effects while 43.75% reported improvements in fatigue and pain, particularly after finishing disulfiram. It is unclear from this study whether disulfiram truly had any beneficial effects on these patients or if the effects could be contributed to antibiotics alone [21].

An in-vitro study found that Disulfiram on its own was not effective in the eradication of *B. burgdorferi* but was effective in combination with nitroxoline and cefuroxime, which kept bacterial viability to 12.5%. Nevertheless, it was concluded that in light of the significant side effects of Disulfiram, the best step moving forward would be to further examine nitroxoline and cefuroxime as potential therapies for stationary phase *B. burgdorferi* [22].

One case study evaluated the effectiveness of intravenous ketamine in treating PTLDS pain symptoms in a 30-year-old female from Florida. The patient was given ketamine infusions starting at 200 mg, and the dose was increased by 200 mg every day until 800 mg was achieved, continuing for a total of a 10-day course. Although there was an initial decrease in pain, it gradually returned, and the patient underwent a ketamine booster infusion. Overall, the patient was satisfied with the treatment and with the subsequent booster infusions, reported radically reduced pain and cessation of depression and suicidal ideations [23]. However, it is essential to note that this patient’s pain symptoms were refractory to pain management procedures and pharmacotherapies, including opioids, and therefore ketamine may not be an appropriate first-line treatment. Further investigation is required to determine its efficacy in treating PTLDS-associated pain before being used in the overall patient population.

In the search for non-pharmaceutical therapies for chronic Lyme disease, one study explored the practicality and effectiveness of resistance training in patients experiencing persistent symptoms of LD. A group of eight participants, who had a previous LD diagnosis and had been experiencing persisting symptoms for at least three months after that, was recruited. They underwent a supervised four-week resistance exercise intervention and had a significant improvement in their joint pain symptoms, exercise performance, and the number of days they felt healthy and full of energy [24]. This sort of exercise plan may be effectively employed by chronic LD patients. But it is fundamental to acknowledge that the study participants represented a small sample size, consisting of Caucasian, college-educated people, and may have little external validity. The exercise program was also guided by exercise professionals and may not be as successful if performed alone. Nevertheless, this looks to be a promising field of study, and further research may make great strides in the non-pharmaceutical treatment of chronic LD.

## Conclusions

Patients with PTLDS continue to struggle to get an accurate diagnosis, proper treatment, and effective care. Over decades, numerous studies have shown the utility of antibiotic therapy in treating PTLDS patients. However, there are no universal guidelines for the diagnosis or a therapeutic regimen to effectively treat PTLDS patients. This review evidences discrepancies and showcases a vast divide in clinical study findings, including no efficacy, short-term efficacy, and long-term benefits of antibiotic therapy. In the future, emphasis should be placed on developing universal diagnostic criteria for PTLDS, which includes assessing symptoms and performing all relevant laboratory tests, before rendering the appropriate antibiotic treatment.

The key points indicated by this review are: global diagnostic criteria for PTLDS do not exist; there is no standardized treatment regimen for PTLDS; studies have focused only on PTLDS treatment without considering proper diagnostic criteria; PTLDS clinical trials are not uniform and their findings are difficult to apply in clinical practice; future research should establish universal diagnostic criteria before conducting treatment trials.
